# In Which Patients and Why Is Laparoscopy Helpful for the Impalpable Testis?

**DOI:** 10.1155/2022/1564830

**Published:** 2022-09-30

**Authors:** Alfonso Papparella, Giuseppina Rosaria Umano, Mercedes Romano, Giulia Delehaye, Salvatore Cascone, Letizia Trotta, Carmine Noviello

**Affiliations:** Pediatric Surgery Unit, Department of Women Children General and Specialist Surgery, Campania University “Luigi Vanvitelli”, Naples, Italy

## Abstract

Since laparoscopy has been proposed in the management of the nonpalpable testis (NPT), this technique has been widely diffused among pediatric surgeons and urologists, but its application is still debated. We conducted a retrospective review to highlight how diagnostic and surgical indications for laparoscopy are selective and should be targeted to individual patients. From 2015 to 2019, 135 patients with NPT were admitted to our surgical division. Of these, 35 were palpable on clinical examination under anesthesia and 95 underwent laparoscopy. The main laparoscopic findings considered were: intra-abdominal testis (IAT), cord structures that are blind-ending, completely absent, or entering the abdominal ring. The patients' mean age was 22 months. In 48 cases, an IAT was found, and 42 of these underwent primary orchidopexy while 6 had the Fowler–Stephens (FS) laparoscopic procedure. Of the first group one patient experienced a testicular atrophy while two a reascent of the testis. In the FS orchidopexy group, one patient had testicular atrophy. Cord structures entering the internal inguinal ring were observed in 35 children, and all were surgically open explored. In 3 cases of these, a hypotrophic testis was revealed and an open orchidopexy was executed. In the remaining the histological examination revealed viable testicular cells in four patients and fibrosis, calcifications, and hemosiderin deposits in the others. Eleven patients presented with intrabdominal blind-ending vessels and one a testicular agenesia. A careful clinical examination is important to select patients to submit to laparoscopy. Diagnostic laparoscopy, and therefore, the anatomical observation of the testis and cord structures are strictly related to develop a treatment plan. In IAT, many surgical strategies can be applied with good results. Laparoscopy offers a concrete benefit to the patient.

## 1. Introduction

The advent of minimally invasive techniques has improved the surgical approach for a wide range of diseases, especially in children, and laparoscopy has undergone significant development over the last few years. Notably, the application of laparoscopy has proved very beneficial in the diagnosis and treatment of nonpalpable testis (NPT) [1, 2]. Approximately 20% of patients with cryptorchidism have an NPT [2]. A testicle is defined as being nonpalpable when located in the abdominal cavity at a variable distance from the internal inguinal ring, in the inguinal canal with a different degree of development, or even absent/or ectopic [2–4]. It is also possible that the testis may have disappeared in utero (a condition known as “vanishing testis,” in which the cord structures end blindly) due to the torsion or vascular accidents that occur during the testicular migration [3, 4]. Patients with bilateral NPT and clinical signs of abnormal sexual differentiation (i.e., hypospadias) require both endocrinological and genetic evaluation [4, 5]. We think that laparoscopy facilitates the diagnostic and surgical strategies for NPT, although such procedures are selective and should be targeted to patients on a case-by-case basis [3]. Therefore, clinical examination is particularly important in these patients, and it must be repeated several times even during anesthesia before surgery. In an effort to locate an NPT, or to confirm its absence, a range of diagnostic tests have been and/or could be used, including tomography computed (TC) scan and magnetic resonance imaging (MRI). Ultrasound (US) can also be useful in verifying the location, morphology, and eventually a remnant of the testis [6]. Each of them has false positives and negatives and is dependent on the ability of the radiologist. Consequently, laparoscopy is the most effective procedure in establishing the fate of NPT and in planning surgery for IAT [3, 4]. In the clinical approach to NPT, it is important to consider patient selection: when an atrophic remnant associated with contralateral testicular hypertrophy (more than 2 cc) is found [7], many authors agree that injury to the testis happens during its descent to the inguinal canal and scrotum [8, 9]: therefore, in these patients, laparoscopy could be unnecessary. When repeated clinical examinations are not able to properly select patients, laparoscopy is always indicated. If laparoscopic finding reveals spermatic vessels and vas that enter in an open inguinal ring, the inguinal exploration is mandatory [10]. In patients with a condition referred to as “peeping testicle,” in which it faces the internal inguinal ring, laparoscopy offers us the possibility of performing a laparoscopic orchidopexy; this is as effective as the inguinal approach [11]. Other surgical strategies may be applicable when laparoscopy reveals a high intra-abdominal testis (IAT) [2, 3, 12–15]. Therefore, it is possible to distinguish two levels of laparoscopic management: diagnostic and therapeutic, which are each other-related.

The aim of this study is to highlight the importance and the close relationship between the patient with an NPT, the laparoscopic finding, and the surgical treatment.

## 2. Materials and Methods

**Figure 1:**
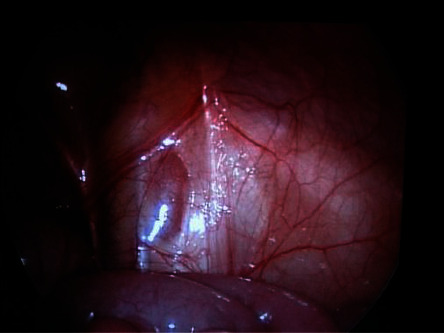
Laparoscopic image of right vanishing testis.

**Figure 2:**
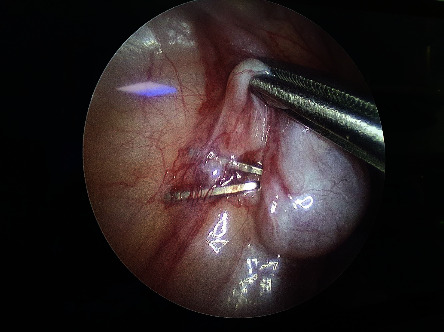
Laparoscopic image of Fowler–Stephens procedure for left intra-abdominal testis.

From January 2015 to December 2019, all the children with NPT presented to the Pediatric Surgery Unit of the University of Campania Luigi Vanvitelli were enrolled for the study. The length of follow-up was 6–18 months. There were no exclusion criteria. The study was conducted according to the Declaration of Helsinki. The Ethical committee of our university approved the study (0007953/I, 04/06/2020). A written informed consent was obtained before the procedure. In all the patients with bilateral NPT, in conjunction with a pediatric endocrinologist, a complete evaluation was obtained, such as Mullerian inhibiting substance level, HCG stimulation, LH, FSH, Testosterone, and androstenedione levels. The hormonal treatments using human chorionic gonadotropin (CHG) or gonadotropin-releasing hormone (GnRH) to induce testicular descent have not been advised because of low success rates and lack of evidence for long-term efficacy [16–18]. All the patients were examined under anesthesia to confirm NPT. If not, these patients were approached by an open orchidopexy. Laparoscopy was performed with the patient placed in the Trendelenburg position to lift the pelvis and to mobilize the bowel cephalad. A 5-mm trocar was then introduced into the umbilicus with an “open” approach, which avoids any damage to intra-abdominal organs [2]. Pneumoperitoneum was then established according to the child's weight. The iliac areas, the pelvis, and the inguinal rings are carefully explored. In the case of intra-abdominal blind-ending cord structures, laparoscopy is adequate to stop the procedure (Figure 1). If laparoscopy reveals an IAT, depending on the distance of the inner inguinal ring (±3 cm), it is categorized as high or low. In addition, the mobility, the length of the spermatic vessels, and the state of the inner inguinal ring are carefully examined to have appropriate information for the surgical management of the patient. In the high IAT, a laparoscopic two-step Fowler–Stephens (FS) orchidopexy is achieved with two additional 5 mm trocars [9, 12] that are positioned in the left and in the right lower quadrant at the level of the umbilicus, lateral to the rectus muscle. In the first stage, a minimum dissection is performed around the spermatic vessel as high as possible, for double clipping or ligation (Figure 2). In stage two, four-six months later, a broad dissection is made in the area around the spermatic vessel, in order to respect the peritoneal vas pedicle necessary for collateral testis blood supply: this is also obtained by minimal use of cautery. When the testicle and peritoneal pedicle are entirely free, a transverse incision is made at the base of the ipsilateral scrotum. A subdartos pouch is created and a grasping forceps are then, introduced into the abdominal cavity and the testis is brought down into the scrotum through the inguinal canal or the medial reflection of the umbilical ligament (Prentiss maneuver). In the lower IAT, a laparoscopic primary orchidopexy is provided using two additional 3 mm trocars. We begin by making an incision along the anterior side of the open vaginal duct if present. Then, the testis is retracted into the abdomen and the gubernaculum testis is divided. The peritoneum surrounding the spermatic vessels is medially incised and its posterior attachments are released using a minimal cauterization in order to respect as much as possible the vascularization of the testis that is carried in the scrotum as in the FS orchidopexy. We do not perform the closure of the inner internal ring and trocars are always extracted under direct visual inspection.

**Figure 3:**
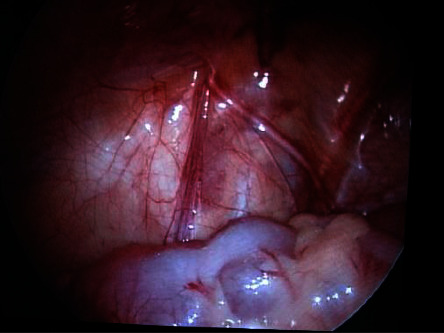
Laparoscopic image of left inguinal ring showing well-developed spermatic vessels and vas entering the inguinal canal.

In the case of spermatic cord structures entering the inner inguinal ring, the size and development are carefully examined and compared to the normal side, if appropriate, as well as to the opening of the internal ring (Figure 3). In most of these patients, when the internal ring was open and/or a normal vascular bundle was present, an open groin exploration was performed after laparoscopy. All specimens removed on inguinal exploration were sent for histological study. If spermatic cord structures were not found, an abdominal laparoscopic exploration was performed till the source of spermatic vessels, and the testicle was regarded as completely absent. The follow-up included clinical examination every 6 months until 18 months after surgery and ultrasound in patients treated with a FS procedure.

## 3. Results

A total of 130 children with NPT were included in the study. The children had a mean age of 22 months (range 9–38 months) at surgery. Sixty-six (50.8%) children had right-sided NPT, and 13 (10%) were bilateral. Among these, 35 (26.9%) patients showed a palpable testis on clinical examination under anesthesia for which an open orchidopexy was performed. Therefore, 95 children underwent laparoscopy according to the procedure described above. In 35 children (36.8%), the laparoscopic findings were spermatic cord structures entering the internal inguinal ring; 48 (50.5%) cases of IAT; 11 (11.6%) vanishing intra-abdominal testes; and 1 case of testicular agenesis. All 35 children with spermatic bundles emerging from the inguinal canal underwent surgical exploration: in 3 cases, a hypotrophic testicle was found and an orchiopexy was performed; in the others, a testicular nubbin was found and removed for histological examination; viable testicular cells (without germ cell elements) were found in 4 patients; fibrosis, calcifications, and hemosiderin deposition in the remaining patients. No malignancy was recorded. In 42 of the 48 cases with IAT, a laparoscopic orchidopexy was performed; one patient experienced a testicular atrophy and two patients had a reascent of the testis; in the last, a redo orchidopexy was performed after 6 and 8 months, respectively. In six patients, due to the high position of the testis, a two-stage FS procedure was performed: one patient had a high bilateral IAT, for which the FS procedure was asynchronous. At follow up, one patient managed with the FS procedure presented with testicular atrophy. The remaining were in scrotal position, ultrasound showed normal morphology but hypotrophy (20%) compared to the contralateral side. There was no difference in hospitalization, and early surgical complications were not recorded in all patients except an umbilical granuloma.

## 4. Discussion

**Figure 4:**
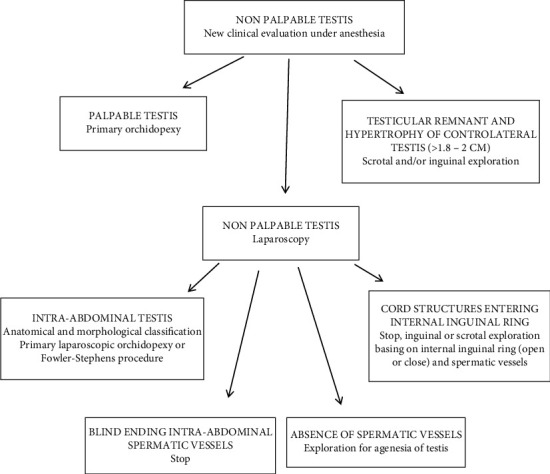
Flow chart showing the management of nonpalpable testis.

Laparoscopy has been widely accepted not only as a diagnostic but also as surgical tool. The clinical selection of the patient and the laparoscopic diagnosis are fundamental for the surgical strategy. The guidelines stress first the importance of laparoscopy in the diagnosis and in the treatment of NPT [16, 18]. Therefore, the data obtained from the laparoscopic diagnosis are essential to accurately address the surgical process and prepare an appropriate management plan for patients with NPT. Nevertheless, the clinical selection of patients must be accurate: in our series of 130 patients, 35 (26.9%) showed a palpable testicle under anesthesia. This may be due to patient movements during the visit or abundant adipose tissue in the inguinal fold. There are several laparoscopic findings that can be encountered during diagnostic exploration [3]. First, one might encounter an intra-abdominal blind-ending spermatic bundle (Figure 1). Second, laparoscopy could reveal an IAT (Figure 2) that can be classified, according to its mobility grade, length of spermatic vessels, and its location, as either high or low, depending also on the distance from the internal inguinal ring (±3 cm) [11–13]. In the case of IAT, the testes could be localized in the iliac fossa, inlet, and deep pelvis in a position lateral to the bladder, or the testes could be “peeping” (facing the inguinal canal). Third, it is possible to note the complete absence of spermatic cord structures (agenesis). In these rare cases, a complete laparoscopic exploration is indicated, till the source of spermatic vessels from the lower renal pole to the internal inguinal ring. In addition, it is possible to encounter spermatic cord structures coming into the internal inguinal ring. In these patients, the size and development of the spermatic vessels should be carefully considered and compared with that of the opposite side, if present, with particular attention to the internal ring aspect [2, 3, 9, 10]. Finally, it is possible to identify a spermatic bundle that crosses the midline (referred to as a crossed testicular ectopia). In the surgical management of IAT, several factors should be considered (Figure 4): the patient's age, the distance of the testis from the internal inguinal ring (<3 cm or >3 cm), the mobility of the spermatic vessels, the patency of the internal inguinal ring, and the grade of testicular development [11, 13]. If the testicle emerges near a patent and/or at a distance less than 3 cm from the inguinal ring, we may choose a standard inguinal and/or laparoscopic orchidopexy.

Generally, IAT is considered to be high when localized in the iliac fossa, or in the passage to the pelvis, or in any case in which the testicles are located more than 3 cm away from the internal inguinal ring. Testes located deep in the pelvis, laterally to the bladder, generally, have spermatic vessels that are mobile and can be easily lifted from their lateral retroperitoneal course: in these cases, the testis could, in our opinion, directly be brought down into the scrotum by the Prentiss maneuver. Nevertheless, this is not the case for testes that are located in the iliac fossa, which usually have short vessels and are often poorly developed. In these cases, a laparoscopic FS procedure could be performed [12, 13]. Generally, this technique, which causes spermatic vessel interruption, can be applied in 20–30% of IAT [13], as also highlighted in our case series. The laparoscopic FS technique represents a valid surgical choice in high intrabdominal testis with high incidence of complications such as atrophy and/or reascent in the inguinal canal no different than other techniques. Based on our previous case series, we consider that laparoscopic surgical skills and the anatomical position of the testis in the abdomen are important factors in deciding the appropriate surgical technique. The laparoscopic FS procedure has to be planned before, at the moment of the laparoscopic observation: it is not a salvage technique. Nevertheless, based on our experience and others', the incidence of atrophy and retraction is, respectively, 12 and 4% for two steps of FS orchidopexy [13, 18]. In order to reduce complications, studies have shown that surgery with the intent of sparing gubernaculum testis reduces the incidence of atrophy [18]. For testicular retraction one study reported an increased incidence when the patent processus vaginalis is closed in the absence of an evident hernia [18]. Furthermore, in the last years Shehata and others authors have proposed a laparoscopic technique where the spermatic vessels are elongated by fixing the IAT at the contralateral side of the abdomen with a mean follow up of 16 months. This technique can also be considered as an option since some studies have validated its safety and effectiveness with the important advantage of preserving spermatic vessels [14, 15].

When laparoscopy reveals blind-ending cord structures (vanishing testis), it is not necessary to perform any other surgical procedure. One specific characteristic of such patients is that the deferential artery is tortuous and serpiginous prior to finishing blindly (Figure 2) [3]. It is this particular group of patients that benefits the most from laparoscopic exploration. In the context of spermatic cord structures that enter the internal inguinal ring, we must consider the following factors: the spermatic vessel appearance (development and collateral circulation), the patency of the internal inguinal ring, and the presence of chromosomal abnormalities. In these patients, it is debated whether to explore the inguinal canal or not [2, 3, 9, 19, 20]. In our series, all the patients with emerging vas and vessels from the internal inguinal ring underwent an inguinal surgical exploration, and histological examination revealed testicular cells only in 4. The main reason for recommending surgical exploration is that a residual gonadal structure, if present, may develop a neoplastic transformation in the long term. However, the very low presence of viable testicular cells (8–10%) in such cases makes this possibility very remote. In particular, Nataraja and coll. in a systematic review regarding the presence of viable germ cells in remnants concluded that there is no solid evidence to establish that its routine excision is indicated even in an inguinal or scrotal position [20]. Laparoscopic observations, along with anatomical and pathological findings (such as calcifications and atrophic residual tissue), support the hypothesis that the disappearance of the testis occurs as a result of a vascular accident during the descent of the testicles into the inguinal canal under hormonal control [19, 20]. The existing literature clearly highlights the importance of the anatomical aspect during surgery when investigating the spermatic vessels and the internal inguinal ring. The previous studies report that hypoplastic spermatic vessels and a closed internal inguinal ring are characteristic features of a missing testicle in the inguinal canal [9]. It is, therefore, crucial that we consider inguinal exploration for patients who have normal spermatic vessels and an open inguinal ring [2, 3], and when chromosomal abnormalities are present [21]. Cases involving chromosomal abnormalities are more likely to have a genetic predisposition to malignant transformation [4]. Furthermore, laparoscopy could be very useful in rare cases, such as IAT torsion [22], splenogonadal fusion [23], and intra-abdominal abnormalities in the major ducts of the testes [24].

## 5. Conclusions

We consider laparoscopy as an effective method for the diagnosis and treatment of NPT [2–4, 16, 18]. There are multiple advantages of using this technique: images can be magnified and enhanced via the “zoom effect” provided by the camera; there are faster postoperative recovery and better cosmetic results. However, the most important advantage of laparoscopy is the possibility to select the best surgical strategy for the patient with negligible incidence of complications [13]. Nevertheless, the direct observation of the testis and of the cord structures allows us to strictly relate the laparoscopic findings to the surgical treatment.

## Data Availability

The data used to support the findings of this study are available from the corresponding author upon request.
